# ATTACHMENT SECURITY AND DEPRESSIVE SYMPTOMS IN OLDER-ADULT MARRIAGES

**DOI:** 10.1093/geroni/igz038.3368

**Published:** 2019-11-08

**Authors:** Sumaiyah U Syed, Joan K Monin

**Affiliations:** 1 Yale University, New Haven, Connecticut, United States; 2 Yale School of Public Health, New Haven, Connecticut, United States

## Abstract

Attachment theory emphasizes attachment security, providing and receiving communication of safety and emotional support, as one of the most fundamental needs in close relationships across the lifespan. Having an insecure attachment style, anxious or avoidant attachment, has been related to depressive symptoms in mostly young adult marriages. This study examined the interpersonal associations between attachment anxiety, attachment avoidance, and depressive symptoms in 98 older adult couples, using self-report measures. The Actor Partner Interdependence model was used to analyze the data. Results show that one partner’s anxious attachment was significantly positively associated with their own greater depressive symptoms (β=2.10, p=0.000). This effect was stronger for husbands than for wives (β=1.13, p=0.002). Results remained when controlling for age, physical functioning, marital length, and socio-demographics. There were no significant cross-partner associations. Findings suggest that attachment anxiety may be particularly impactful for depressive symptoms in husbands.

